# Is Democracy Therapeutic? A Deweyan Reading of the Institutions of Antipsychiatry

**DOI:** 10.1007/s12124-021-09639-3

**Published:** 2021-08-27

**Authors:** Luis S. Villacañas de Castro

**Affiliations:** Department of Language and Literature Education, Facultat de Magisteri, Universitat de València, Ave. Tarongers 4, 46022 València, Spain

**Keywords:** John Dewey, Democracy, Antipsychiatry, Institutions, Dialectics

## Abstract

This article presents a Deweyan reading of the processes of critique, experimentation, and reform that took hold of a minority of psychiatric institutions in Western Europe during the nineteen-sixties and seventies, under the influence of the so-called Italian and British *antipsychiatry* movements. Framed within a specific understanding of the sixties, the article examines these complex theoretical and institutional operations against the background of John Dewey’s idea of *democracy*, which it interprets, above all else, as the constant provision of material, intellectual, and human resources for the people to directly transform their environment and themselves in increasingly complex and creative ways. After acknowledging the historical and conceptual discontinuities that exist between these two autonomous bodies of knowledge, the first section presents a summary of Dewey’s philosophy. Next the article sheds light on Basaglia’s and Laing’s antipsychiatric projects by interpreting them as a sustained effort to distinguish between schizophrenia as a *first* and a *second* disease, an epistemological search in the midst of which each of them ended up creating new institutions that necessarily embarked their inmates on a radical process of Deweyan growth. The key role of the sixties counterculture is emphasized at this point, and examples from Gorizia’s and Trieste’s asylums, as well as British community households, are read in terms of Basaglia’s and Laing’s *negative* and *affirmative* dialectics, respectively. Finally, in the last two sections, the article argues that antipsychiatry’s analysis of psychotic behavior significantly enlarges Dewey’s understanding of the circuit of growth and experience, and that Dewey’s ideas of growth and experience provided, in turn, a missing criterion for defining mental health and deriving coherent therapeutic and institutional concretions.

## Introduction

This article presents a Deweyan reading of the intense processes of critique, experimentation, and reform that took hold of a minority of psychiatric institutions in Western Europe during the nineteen-sixties and seventies, under the influence of the so-called *antipsychiatry* movement (Burns, 2020; Double, 2002; Foot, 2015; Wall, 2019). It is framed within a larger interpretation of the sixties which looks at the events of the decade from the standpoint of John Dewey’s philosophy. Through a close analysis of socio-economic, political, and cultural phenomena of the period—radical ones included, like the upsurge of hippie communes in North America (Villacañas de Castro, forthcoming), or the institutions of antipsychiatry examined in this case—this historical interpretation takes the view that the aspirations and forms of life displayed by the sixties counterculture were not peripheral manifestations from radicalized, eccentric minorities. Rather, they were organic expressions emerging from vibrant societies, parts of whose youth were striving to meet society’s full democratic potential in ways that were strikingly similar to how Dewey conceived the widening of the democratic spiral. Dewey was a towering figure in American philosophy, at least until the late nineteen thirties; if justified, the idea that his account of democracy became incarnated in the counterculture of the sixties would involve two things. First, that these countercultural manifestations were legitimate expressions of how democracy expands and revitalizes itself in history. And second, that Dewey’s philosophy (hailed by Sidney Hook as the paragon of North American thought) was more radical that it was and is widely presumed to be.

The present article develops this argument in relation to *antipsychiatry* and its institutions, of which it presents a Deweyan reading. Like Foot (2015), I use this term in a restricted way, to identify the radical and critical approaches to mental illness developed by Ronald D. Laing in England and Franco Basaglia in Italy. For context, I will briefly summarize the main ideas of these radical thinkers and the movements they spearheaded. A unifying trait of their contributions was their effort to analyze, criticize, and modify theoretical and practical concepts which seemed to owe their existence not to proper scientific theory, but rather were manifestations of societal biases which inhered in medical and psychiatric institutions. From this perspective, psychiatry “was regarded as part of the problem” (Double, 2002: 235), to the extent that it formed part of what Laing and Basaglia considered to be a highly problematic society. In accordance with the zeitgeist of the late sixties, in their Introduction to the Spanish edition of Basaglia’s *L’istituzione negata* (which I have used, in the absence of an English translation) García et al. (1970: 17) emphasized that “in a repressive, oppressive, and police system, psychiatry must also be repression, oppression, and police.”

This article examines these operations of conceptual and institutional critique, reform, and experimentation against the background of Dewey’s philosophy, especially against his idea of *democracy*—possibly the one central concept in his work and guiding thread of his multidisciplinary oeuvre. In line with Westbrook’s (1992) intellectual biography of the Vermont philosopher, this article interprets Dewey’s idea of democracy, above all else, as the constant provision of material, intellectual, and human resources for people to directly intervene in and transform their environment and themselves. By reading this notion side by side with the main tenets of antipsychiatry, I intend to force these two conceptual realities into a dialogue through which each can shine its light on the other’s strengths and frailties. This dialogue will unfold in the following way: After acknowledging the historical and conceptual discontinuities that exist between these fully autonomous bodies of theoretical and practical knowledge, the next section presents a summary of Dewey’s philosophy, with a special focus on his psychology and how he believed his democratic project could be realized in and through the institutions of society. Next, in section three, the article will shed light on Basaglia’s and Laing’s projects by interpreting them as sustained efforts to distinguish between schizophrenia as a *first* (biological) and a *second* (institutional) disease, an epistemological search in the midst of which each of them ended up creating new institutions that necessarily embarked their inmates on a radical process of Deweyan growth. At that point the article will bring to the fore the strong ties that both the Italian and British strands of antipsychiatry shared with the sixties and seventies counterculture, which I interpret as instrumental to their breakthroughs. By reading these authors, works, concepts, and realities together, I wish to argue that antipsychiatry was, in fact, essentially Deweyan—and that likewise, had Dewey offered his own systematic psychiatry, it would have been one aligned with the fundamental tenets of antipsychiatry. The article brings this reciprocal relationship to bear on the question of democratic institutions and their therapeutic quality, as understood in a broad sense. As is shown in the last two sections, antipsychiatry’s take on psychotic behavior significantly enlarged Dewey’s interpretation of the circuit of growth and experience, and thus increased the concepts and tools required for democracy to address mental illness. On the other hand, to the extent that Dewey’s ideas of growth and experience were founded on robust democratic principles which anchored in the ontological plane, I believe they can provide a missing criterion for defining mental health and deriving coherent therapeutic and institutional concretions. This is exactly what the title of this article wishes to convey by reformulating one of the main mottoes of the Italian *Psiquiatria Democratica*—“freedom is therapeutic” (see Fioritti, 2018)—in terms of Deweyan democracy being therapeutic. Is democracy therapeutic? The arguments presented in this article conclude that both Dewey and the antipsychiatrists would have agreed and affirmed that it is.

## Dewey and Democratic Institutions

The article first needs to confront the wide historical and conceptual discontinuities that separate the two bodies of theoretical and practical knowledge being compared. While full of rich insights into the processes of human thought, Dewey’s psychology was subservient to his interests in democracy, ethics, and logic; he remained more attentive to mental health than to exploring the roots of mental illness, although illuminating remarks regarded said roots can still be found in his work. At no point was Dewey’s philosophy an influence on any of the theoretical and experimental endeavors of antipsychiatry, neither on its critique of schizophrenia and attack on the mental asylum, nor on its search for alternative institutional forms. Nor did Dewey’s name crop up among the main tenets that were embraced by the movement. Maxwell Jones and Ervin Goffman, along with Karl Marx, Franz Fannon, Primo Levi, Jean-Paul Sartre, even the French surrealists, inspired British and Italian antipsychiatry movements (Foot, 2015; Wall, 2019). Not Dewey. His presence in the primary and the secondary literature on antipsychiatry is very scarce, collateral at most. For example, during his analysis of the institutions of antipsychiatry in terms of “working utopias,” Crossley (1999: 822) discussed Dewey’s notion of *habit* formation, yet his remarks, while pertinent from a theoretical point of view, were peripheral to an argument that always remained framed within Bourdieu’s notion of *habitus*. Likewise, when Laing, Phillipson, and Lee (1966) supported their claim to the mutual interdependence of individuals and their environment by resorting to American pragmatism, Dewey was not among the philosophers named. Finally, Maxwell Jones’ seminal work on therapeutic communities never mentioned Dewey directly (Jones et al., 1953), despite the pragmatist inspiration that Basaglia (1970 [1968]: 134) did not fail to identify in Jones’ model.

These discontinuities notwithstanding, I believe there remain other conceptual coincidences that justify my attempt to establish a dialogue between these two schools of thought. A reconstruction of Dewey’s philosophy seems inevitable at this point, if only to have a concrete set of ideas to contrast against antipsychiatry in the following sections. My account will be selective per force, yet it will hopefully remain truthful to Dewey’s original purposes. Apart from relying on key texts spanning across his early, middle, and later works, my interpretations follow for the most part those of Westbrook’s (1992) massive intellectual biography, *John Dewey and American Democracy*, which I have heavily drawn upon. All of the primary and secondary literature I have consulted stresses the robust conceptual architecture that brings together the ontological, ethical, educational, political, psychological, and aesthetic planes in Dewey’s thought, all of which rests upon a central structure that can be best reconstructed when one identifies—as Westbrook did—the idea of *democracy* as its vault key. Accordingly, the first part of this section focuses on this grand concept, while the second attempts to derive those consequences that are relevant for the article’s later engagement with antipsychiatry.

In the second edition of their *Ethics*, Dewey and Tufts (2008 [1932]: 349) affirmed that “democracy projects to their logical and practical limit forces inherent in human nature and already embodied to some extent in human nature.” In accordance with this quote, what first strikes the reader concerning Dewey’s use of “democracy” is how strongly it connected to the concept “nature,” with which it formed an indissoluble pair. Democracy was a normative ideal that exuded from the basic ontological dynamics that Dewey found in nature and which democracy, in turn, had to purify and translate into human, social, and institutional forms designed to better direct our societies. In *Art as experience*—where Westbrook (1992: 391) found the best rendition of Dewey’s naturalist ontology—these dynamics were described in terms of the “growth” and “experience” of the “life creature” (Dewey, 2005 [1934]: 1). Every single attribute, activity, or quality of the human being—Dewey claimed—was rooted in the “stream of living” (5) that bonded individuals and their environments as interconnected realities. As could be expected, this was also the case with the main concepts in Dewey’s psychology, where the ontological foundation shaped not only his critique of the “reflex arc concept” and its subsumption in the wider circuit of experience (1998 [1896]), but also the central notion of *habit*, a disposition that human beings acquired by interacting with their environment through occupational frameworks whose means and ends were socially mediated (2008c [1922]). Even the mind, Dewey (1917: 271–2) said, “represents something acquired […], a reorganization of original activities through their operation in a given environment. It is a formation, not a datum; a product, and a cause only after it has been produced.”

Habits, growth, and experience were—like democracy—consubstantial to the life creature, since they could not be erased from reality; yet, at the same time, they referred to qualities that could be more or less present in human interactions with the social and natural environment. “It is not enough to insist upon the necessity of experience,” Dewey (2015 [1938]: 27) warned. “Everything depends upon the *quality* of the experience which is had.” Certain habits could likewise be appraised above others, so it would become necessary to create environmental situations which specifically cultivated them, as Hansen and James (2016) emphasized. This perfective quality shared by growth, habits, and experience paved the way for Dewey’s idea of a democratic society and the role of institutions in it, which remained educative at heart. An offshoot of his naturalist ontology, Dewey suggested that any human intervention (institutional or not) that expanded the affordances that individuals naturally found as they interacted in and with their surroundings was to be considered, in itself, *democratic*. The more these affordances were expanded, the more growth they would generate, and the more intense their democratic quality would be. “If society is a social organism,” Višňovský and Zolcer (2016: 57) claimed, “democracy is an expression of its ideal organization.”

These affordances had to do with humans’ unique ability not only to *adapt* to the world, but to *transform* it rationally and in accordance with certain needs, purposes, and aims. As individuals enmeshed themselves into their surrounding environment and became affected by it, they gradually deepened their understanding of the elements and relationships that constituted this situational context, as well as of the place that they—as human beings—occupied inside it. They then tried to give this knowledge a systematic, unified, communicable character, one that would enable other individuals and collectives in the future to expand their own efforts (and hence also humanity’s democratic spiral) at further manipulating their environment in ways that, in turn, continued to empower and emancipate themselves from tyrannies imposed by nature or by other human beings (Dewey, 2012 [1916]: 89; 2016 [1927]: 203). Even more than scientific works, art entailed the “consummation” of experience (2005 [1934]: 36–9), a “fulfilling conclusion” that maximized its communicable character and rendered it useful for other fellow human beings to include it as a resource in their own subsequent experiences and transformations. Finally, these realizations had to be brought to the local communities, where scientific and artistic outcomes of experience acted as mirrors in which human collectives not only recognized themselves as they were but also projected their existence into the future (1962 [1929]: 137–8; 2005 [1934]: 84). All of these moments were constitutive of the ever-expanding, ever-empowering spiral that best illustrates the organic development of growth and democracy as interrelated concepts.

While democracy’s ontological substratum was consubstantial to natural and social interaction itself—“the clear consequences of a communal life, in all its implications, constitutes the idea of democracy” (Dewey, 2016 [1927]: 176)—the translation of this ontological structure into concrete historical norms, institutions, or artistic and scientific outcomes, was only partially successful, only partially loyal to democracy’s potential, and consequently ridden by tensions and open to improvement. At his most utopian, Dewey conceived of democracy as a “complex, organic work of art” (Westbrook, 1992: 416), yet this was far from always being the case. Through the ontological founding of the life creature, democracy was connected with the possibility of actively transforming the world, yet this possibility depended on individuals having unmediated access to tools and material objects, ideas, and other human beings with whom to communicate and openly and freely share, discuss, and develop their projects. For democracy to fully realize itself, it had to encompass all three elements and extend them to every single domain and mode of human association in society.

Dewey’s ambivalent account of institutions should be emphasized at this point. Straume (2016) and Bernstein (2010: 304–5) criticized Dewey for his “little emphasis on institutional analysis—on what sorts of institutions are required for a flourishing democracy.” This silence can be partially accounted for. Premised as his philosophy was on the need to keep a fluid rapport between individuals among themselves and with their social and natural environment, Dewey (2012 [1916]: 40) often held institutions responsible for limiting this exchange, by establishing “absurd and impossible separations between persons and things” and endangering the democratic project. Still, his discomfort did not give way to the complete dismissal of institutions tout court. At the end of the day, the door remained open for institutions that encouraged flexible and creative habits, rich experiences, and democratic arrangements with the environment. The mission of institutions in a democratic society could be characterized as both reflecting and consummating the interconnectedness that defined the ontology of the life creature through a parallel institutional system. Like habits and customs, institutions “were to be judged according to the degree to which they contributed to the development and qualitative enhancement of associated living” (1973 [1919]: 132). Instead of arbitrarily distancing people from goods, resources, and from each other on the grounds of selfish interests and minority privileges, democratic institutions had to create a veritable and unitary reservoir of social wealth, one that should be open to everyone’s give and take. As Dewey (2008b [1917]) explained in his essay “The principle of nationality,” the existence of different cultures, races, nationalities, social classes, languages, families, sexes—even age-groups—needed not become an obstacle for a flourishing democratic society, so long as these divisions did not become as intense as to bar any other social group from directly accessing and contributing to what was needed to collectively transform their realities. To guarantee this was the role of democratic institutions in democratic societies.

Dewey offered his most detailed analysis of democratic institutions in relation to schools, where the concepts of experience, growth, and habit inevitably acquired an educative quality. His educational writings provide the royal road to a general Deweyan theory of democratic institutions that, once understood, can be extrapolated to non-educational ones (like those of antipsychiatry). In schools, the dynamics of the life creature realized themselves in terms of curricular subject matter, methods of instruction, facilities of the school building and its whereabouts, and lastly also of the students’ accumulated habits and past experiences (Dewey, 2015 [1938]: 28). Schools became democratic to the extent that their teachers were able to mobilize these material, immaterial, and human resources to create situations that allowed children to refine their dispositions and habits by channeling them through the artistic and scientific traditions of inquiry, while simultaneously imbuing them with agency to transform their nearby environment. *Occupations*—“a mode of activity on the part of the child which reproduces, or runs parallel to, some form of work carried on in social life” (2001 [1915]: 83)—were the pedagogical means to achieve this aim. Its realization revolved around teachers that were willing to tap into the potential for growth found in the contexts in which children underwent their daily experiences, but also to erase inside their classrooms the undemocratic constraints that limited the artistic and scientific—educative in the last instance—potential of these activities in their original settings (Dewey, 1962 [1929]: 127–129). “The key to the present educational situation,” Dewey (2012 [1916]: 369) explained in chapter XXIII of *Democracy and education*, “lies in a gradual reconstruction of school materials and methods so as to utilize various forms of occupations typifying social callings, and to bring out their intellectual and moral content.” Through occupations, schools built on the original frameworks of interaction found in a diversity of contexts of Dewey’s American society—mainly homes, neighborhoods, workshops, and factories—and liberated and expanded their scientific and artistic properties by merging them with subject matter. This culminated in the orientation of said properties towards the fulfillment of social aims that would have been previously agreed upon, in this case, by all members of the school community: children, teachers, and parents.

As has been mentioned, the fact that schools were the paradigmatic democratic institution meant that they offered a model for the rest of institutions in society. In the same way as teachers provided the children with an opportunity to creatively interact with their local contexts in ways that enlarged their horizons and increased the depth and complexity of their experience, so the rest of institutions had to distribute the accumulated wealth of humanity among the people and sanction the means and ends that their application should have in society (Dewey, 1935: 61). It is safe to say that, for Dewey, democratic institutions were the *educators* of the entire society: “Every place in which in which men habitually meet—shop, club, factory, saloon, church, political caucus—is perforce a school house, even though not so labeled,” he said (2008a [1912–13]: 303–4; see also 1962 [1929]: 61). This educational role depended on whether institutions made sure that the people’s universal appropriation of society’s resources enhanced their capacity to transform their surroundings in more conscious and sophisticated ways—or, to say it in Dewey’s (2012 [1916]: 90) terms, whether institutions led to a “reconstruction or reorganization of experience which adds to the meaning of experience, and which increases ability to direct subsequent experience.” Those committed to realizing any given social function inside an institution had to do so by reaching out to the local communities, freely communicating with each other and them, and by making concerted use of the ideas, technologies, and products of humanity. Apart from schools, in search for examples of this institutional model, Dewey often referred to Hull House, a *settlement house* that Jane Addams opened in 1889 and with which Dewey closely collaborated during his stint at the University of Chicago. Despite not being an educational institution per se (and despite being shaped by ideas other than his own), Dewey (1902: 84) heralded it as an ideal democratic institution, and thus an example of what he “wanted to see […] every public school doing.” For its ability to reinvent itself into a theatre, a maternal hospital, museum, hostel for working girls, meeting room, auditorium or lecture hall, all to enrich the lives of the poor and oppressed with powerful artistic and scientific resources (Ryan, 1995: 149–53), Hull House is also an antecedent of the democratic drive that characterized the institutions of antipsychiatry, which the next section focuses on.

By contrast, any attempt at fulfilling a social purpose without democracy led to perverted, devalued, and failing institutions, incapable of satisfying their functional aims. This was what Ervin Goffman’s (1991 [1961]) seminal work, *Asylum. Essays on the social situation of mental patients and other inmates*, clearly demonstrated. Together with Maxwell Jones’ description of therapeutic communities, Goffman’s book set the grounds for the subsequent intervention of antipsychiatry along the lines set by Dewey’s work.

## The Institutions of Antipsychiatry: A Deweyan Reading

Unfortunately, restrictions of space prevent me from fully expounding the double transition from Goffman’s (1991 [1961]) characterization of mental asylums as *total institutions* to Maxwell Jones’ model of *therapeutic communities* (Jones et al., 1953), and from the latter to Laing’s and Basaglia’s institutions of antipsychiatry. Were it possible for me to develop this argument entirely, I would try to prove that this double transition was one whereby Dewey’s (2016 [1927]: 175) democratic ideal was “carried to its final limit, viewed as completed, perfected”, were it not—again—for the fact that such an institutional model did not predate antipsychiatry’s realizations, except as utopian intuitions implicit in Dewey’s writings which only the transformative energy displayed by Gorizia’s and Trieste’s asylums, Kingsley Hall, or the rest of Philadelphia Association community households made recognizable as their own philosophical antecedents. They did so precisely by offering compelling instances of human growth. Just as Dewey had made sure that school occupations connected with the democratic strengths of industrial capitalism while simultaneously removing the obstacles to growth posed by that same society, an overarching analysis of Goffman’s total institutions, therapeutic communities, and the institutions of antipsychiatry would reveal how each of these institutions built on the previous outlooks of others by selectively appropriating their more democratic features and discarding the rest.

### First and Second Disease

Basaglia and Laing, too, built on Goffman’s and Maxwell Jones’ models as a springboard towards even more extensive and richer forms of growth, yet not without first purifying the asylums and therapeutic communities of the oppressive elements they had inherited from a society of which they failed to be sufficiently critical. As Wall (2019) has shown, it was thanks to the close ties that Basaglia’s and especially Laing’s circle kept with the counterculture that antipsychiatry was able to enrich, expand, and channel progressive therapeutic perceptions towards artistic consummations that did full justice to the aspirations of Deweyan democracy and growth. Indeed, Cummins (2018) has noted that no rhetorical strategy was more characteristic of the Italian antipsychiatry movement than that of “putting within brackets,” an operation that Basaglia enacted not only in relation to some basic assumptions of psychiatry but, also, to the dominant political, ethical, and economic assumptions of Western society in the sixties. This was also true of Laing who, in line with the tradition of philosophical skepticism in which he has often been inscribed (Mezan, 2015), spoke of a need to “put in parenthesis,” to “bracket off,” to “mind the parenthesis or suspension of judgment,” especially in relation to the diagnosis of schizophrenia and the juridical and political implications that ensued therefrom (Laing & Esterson, 1975 [1964]: 18). Concerning all of these issues, putting schizophrenia within brackets was more than just a rhetorical gesture; it was a methodological strategy justified by what the antipsychiatrists regarded as deep epistemological faults at the core of their discipline. This much was claimed by Basaglia (1985 [1973]: 42–4), Laing (1973 [1967]: 99–100; 1967: 52), and Cooper (1974a [1967]: 25; b [1971]: 59), as had been done by Goffman (1991 [1961]: 309–15) and Jones et al., (1953: 72) before them: namely, that nothing could be known or cured about schizophrenia from the way contemporary mental asylums dealt with it. “The concept of schizophrenia,” Laing said, “is a kind of straightjacket that severely restricts the possibilities both of psychiatrists and patients,” which explained the need to “tak[e] off the straightjacket and see what happens” (cited in Schatzman, 1969: 296). Antipsychiatry aspired to put the entire construct within parenthesis, study the phenomena under a new light, and hopefully create new conceptual, therapeutic, and institutional approaches that would be able to address the human plight that, by all accounts, the theory and practice of traditional psychiatry had brought to a dead end. Let us see how this was done.

In his article in the 1985 special issue of the *International Journal of Mental Health*, Mollica (1985: 30) applied the distinction between *first* and *second disease* to Basaglia’s project, a conceptual pair that I wish to extend to Laing’s contribution. By considering this opposition as the real leitmotiv of both the Italian and British strands of antipsychiatry, the article will present a unified picture of the medical endeavors in the midst of which Laing, Basaglia, and their respective teams succeeded (each in their own way) in concocting alternative institutions capable of harboring forms of Deweyan growth that, at the same time, were considered therapeutic. As a matter of fact, the distinction between first and second disease was the implicit (for Laing) or explicit (for Basaglia) conceptual counterpart of the methodological “bracketing” that both of them applied to the traditional readings of schizophrenia. The argument unfolded as follows: When understood as a first illness, schizophrenia implied a series of symptoms whose cause—whether biochemical, neurophysiological or psychological (Gordon, 2016 [2010]: 9)—lay entirely within the individual (Laing, 1973 [1967]: 99). The problem for antipsychiatry was that yonder internal cause remained unknown and hence the patients basically remained untreated. As Goffman (1991 [1961]) exposed, together with the under-resourced and understaffed condition of mental hospitals, the epistemological shortfalls that plagued the notion of first illness turned asylums into custodial rather than medical premises. Pressed by the legion of human casualties that resulted from this paralysis, Laing and Basaglia posed the alternative hypothesis that the nature of schizophrenic symptoms was neither biochemical, neurophysiological nor psychological, but derivative of a second illness whose roots were *social* and *institutional*—that is, *secondary*. “The revolutionary step of anti-psychiatry,” Double (2002: 232) declared, “was the conviction that society and the family themselves are directly pathogenic.” The two institutional realities to which this understanding was applied were the *mental asylum*—in Basaglia’s case—and the *family*, by Laing’s and British antipsychiatry in general.

In *Individualism: old and new*, Dewey (1962 [1929]: 132) had explained madness as the result of an individual “withdraw[ing] from reality into a merely inner world.” In Dewey’s terms, this operation led to a drastic reduction in the range of interaction and habit formation that the life creature could engage with in his or her social and natural environment, and also to the impoverishment of the growth it was able to enjoy. Both the family and the mental asylum precipitated similar processes, according to antipsychiatry. In the mental asylum, for example, *emerginazione*—social exclusion through institutionalization, in English—was the process responsible for this secondary illness (Jervis, 1985 [1968]). Based on the analysis of Nazi and fascist concentration camps carried out immediately after the Second World War (Foot, 2015: 95–106), *emerginazione* implied dehumanizing patients through chronification, isolation, and internment. This operation was socially, culturally, and economically qualified to the extent that it was exercised upon the most underprivileged strata of the working classes; actually, it was only conceivable precisely because it was carried out on people who were already oppressed, defenseless, and disenfranchised (Basaglia, 1970 [1968]: 139–45; Basaglia & Basaglia Ongaro, 2018 [1966]: 125). In the family, on the other hand, schizophrenia as a secondary illness related to children being exposed, from their tender age, to interactions that—like the description offered by Bateson et al. (1956) of double-bind statements—not only *invalidated* their subjective experience but also *mystified* and dis-acknowledged this invalidation even as it was taking place (Laing, 1973 [1967]: 31; Laing & Esterson, 1975 [1964]). The child was thus bereft of any ground on which to build his or her solid self, and had no option but to exist amidst a horizon of “ontological insecurity” (Laing, 1990 [1960]). This alienating or maddening process can be translated into Dewey’s philosophical terms the moment schizophrenia is understood as the result of the “live creature” being forced into “untenable positions” (Laing, 1964), “unlivable situations” (1973 [1967]: 95) within a familial environment that, whilst temporally and spatially continuous, was experienced as inconsistent in terms of the nature and the meaning of the relationships that were made in it. This contradictory and erratic character collapsed upon the immature self of the family member who, to use Tomasini’s phrasing (cited in Foot, 2015: 42), lacked the immaterial or inter-subjective resources to disentangle the Laingian knots that were bounded around him or her.

From this angle, antipsychiatry was a critical effort to drain whatever explanatory power remained in the concept of schizophrenia as first illness and refocus that power towards those two institutions of society. If confirmed, this hypothesis would open new paths for active treatment that, as Goffman (1991 [1961]: 332) and Jones et al. (1953: 159) had made clear, were scarcely present in the asylum. Although neither Laing nor Basaglia banned the possibility that a first illness existed—something that has not always been understood—they decided to place that notion within brackets and render it ineffectual until each of them, through their separate yet parallel projects, had made sure that the symptoms of schizophrenia could not be explained otherwise. In his contribution to *The negated institution*, for example, Basaglia (1970 [1968]: 146) likened this epistemological endeavor to gradually peeling an onion, to removing the institutional layers of madness to see whether the first illness finally appeared at the core. Yet in the end Laing and Basaglia tentatively concluded that there was no core, only layers upon layers of institutional secondary illness, pushed and fossilized together under the weight of tradition and medical blindness (Basaglia, 1985 [1973]: 51).

No matter how far antipsychiatry pushed its exploration into schizophrenia, the notion of first illness always receded farther back into the horizon. As a result, Laing’s and Basaglia’s methodological and therapeutic dimensions also remained anchored to this secondary, institutional plane. For humanitarian, epistemological, and therapeutic reasons, antipsychiatry believed in the need to have *new* institutions that were completely different from those in existence (Laing, 1967; McGeachan, 2014; Ognaro Basaglia, 1985: 10–11). Inside them, traditional psychiatric operations such as individualized *diagnosis* and *therapy*, for example, would not be conducted, as was noticeably the case in the hospitals that Basaglia directed (Bennett, 1985: 80–1; Cummins, 2018) or in the community households of the Philadelphia Association that Laing co-founded in 1965 (Chapman, 2020a; Laing, 1967; Thompson, 2015). For one, Leon Redler said, the latter houses had “no one to make and no context in which to make a psychiatric diagnosis. There was no binary system of patients and staff, surely no formal one” (cited in Gordon (2016 [2010]: 21)), thereby underscoring that the Laingian households had already pulled down this undemocratic barrier (see also Fussinger, 2011). On the contrary, the therapeutic endeavors of antipsychiatry revolved around the Deweyan notion that the institution’s social and material environment would cater to patients’ richer and more engaging interactions, and that this would bring forward positive inward changes. In a similar way to how educators—according to Dewey—could not “directly reach inside a student’s mind or heart and rearrange the internal wiring,” but rather had to “teach indirectly, through the medium of the environment [they] set up” (Hansen & James, 2016: 201), Laing and Esterson planned to create a social milieu that, “unlike a mental hospital or similar institution […], will be adapted to the patient, and not the patient to the milieu” (MS Laing A627).

However, premised as Laing’s and Basaglia’s projects were on different explanations of schizophrenia as a secondary illness (and on different philosophical traditions that accounted for them), they also conceived of the new institutions differently. For Basaglia, neither mental illness nor the asylum (its institutional counterpart) could be disconnected from the wider dialectics of the class struggle, an association that resulted in the view that, no matter how democratic and creative the transformations brought on to the hospitals of Gorizia’s and Trieste, they should be conceived only as transitory steps to the hospitals’ closure—the only logical endpoint of his de-institutionalization project. Burns (2019: 21), at this point, was categorical: Basaglia “set out to abolish the mental hospital, not to reform it.” Just as the assemblies, elections, and workshops in the said hospitals never failed to emphasize how the inmates’ status, as mental patients, was the ultimate link of a wider chain welded in poverty and oppression; and just as the inmates’ realization of this bigger picture constituted the real therapeutic moment (Basaglia, 1970 [1968]: 154; Basaglia & Basaglia Ongaro, 2018 [1966]: 121), so Basaglia’s institutional actions were guided by the belief that the mental asylum had to dissolve itself for schizophrenia to return back to society, where it would be openly and fully experienced with the rest of the elements of the class struggle (Jervis Comba, 1970 [1968]: 235; Mollica, 1985: 36). No wonder, then, that the main legislative outcome that stemmed from the Italian antipsychiatric movement—Law 180, passed in 1978—enforced the closure of all of the mental asylums and their replacement with only the minimal infrastructure needed to sustain patients, families, and communities in moments of critical need (Bennett, 1985).

This new vision set Basaglia apart from the endeavors associated with the therapeutic community movement. Inspired by Jean Paul Sartre’s philosophy, Basaglia’s contribution to *The negated institution* mapped a *negative dialectics* according to which democratic affirmations within asylums would only be effective if they simultaneously involved their radical self-negation as part of a class-ridden society that had to be ultimately confronted. This negative, self-reflective move was what, according to Schittar (1970 [1968]), therapeutic communities most lacked, and hence the reason why they had stagnated into a concrete, technical model like Maxwell Jones’. While Basaglia’s hospitals also went through phases of communitarian democratic transformation, these were but transitory moments that the institution soon left behind to further its process of negation and dissolution, one that would allegedly give rise to more fundamental contradictions with society (Basaglia, 1970 [1968]: 151). “There “[could] be no balance or stability until all the wards have been opened,” Basaglia said at one point. “At the time being, there still remains a ward to be opened, and this allows us to continue negating. After this, it will be necessary to look for other forms of negation that allow us to negate practically everything” (as cited in Jervis Comba, 1970 [1968]: 238).

On the other hand, Laing’s notion of democracy was less influenced by proletarian emancipation than by Donald Winnicott’s notion of the authentic, creative self (Kirsner, 2015: 71–2), one which Laing alternatively enriched with countercultural and—after 1970—mystical or spiritual possibilities (see Sedgwick, 1971). As Chapman (2014: 22) said, “Laing’s conception of liberation is very much an individual one.” The essential factor was that Laing’s idea of schizophrenia was not bound to the class struggle but to the alienating patterns of communication that took place inside the nuclear family, an institution that—maybe due to its revered place in Italian culture—Basaglia did not problematize in any fundamental way. This is not to say that Laing's and Cooper’s ideas on the family were without social ramifications. According to both, the “power” of the familial institution as a “social mediating function” (Cooper, 1974a [1967]; b [1971]: 6–7) resided in that its pathological intersubjective patterns were the same ones that most institutions followed in their own interactions (Laing, 2015 [1968]). This meant that the familial communication model was first interiorized by each individual and was later instrumentalized by institutions acting on all levels of society—including the school and the asylum—in order to “exercise the power of the family over those who engage with [them]” (Wall, 2019: 159). In terms of liberation, so long as Kingsley Hall or the rest of the Philadelphia Association community households incarnated clear alternatives to the alienating forms of communication forged within, and later diffused outside, the family, they fulfilled a liberating and healing function.

### Basaglia’s Negative Dialectics

For all their conceptual discrepancies, the truth is that democracy looked very much the same in the “negated institutions” of Psychiatria Democratica as in the British households or “anti-hospitals,” as Cooper (1965) once called them. Both were institutions whose perception of mental health in many ways paralleled Dewey’s vision of enriched life and growth, and whose therapeutic approaches also seemed to embrace the mission that Dewey had originally attributed to democratic institutions: namely, to guarantee individuals’ direct access to the material and immaterial resources and expanded human networks that would allow them to act artistically upon their worlds. As can be expected, in accordance with Sartre’s idea that freedom could only flourish in the void opened up by negativity, Basaglia inscribed any widening of vital possibilities within the operation of the system’s dismantling. In quite literal terms, democracy had to flourish amidst the ruins of the institutional edifice. The asylum could only be affirmed while it was being closed down or negated, since this was the paradoxical precondition for the inmates to appropriate it in a democratic way. As Foot (2015) described in his chapters devoted to the Gorizian experience and the late transformation of San Giovanni asylum in Trieste, these two institutions made creative use of resources and of expanded human networks that coincided with the opening of concrete wards inside the buildings, with the demolition of walls that separated women from men, or with the final removal of the asylums’ front gates. As had been the case with the Italian schools at Reggio Emilia at the end of the Second World War, it was among the crumbling blocks of brick, cement, and plaster that the inmates of these two asylums found the resources they needed to pursue renewed vital projects which would no longer be confined, physically or spiritually, to the perimeter of the mental hospital. Similarly, it was only through the sequential opening of the asylum’s wards that the inmates and staff were to strengthen the relationships among themselves (in and through assemblies, workshops, or projects like running a bar or publishing *Il Picchio*, the asylum’s newspaper), or to establish new lines of communication and collaboration with the rest of society. So was an alternative institution created with, and on, the ruins of the old one. Basaglia (1980: 23) put it in the following terms: “Social awareness filters through the walls into the asylum, on the one hand, as a search for increased contacts and democracy in social relationships; and on the other, finds its way out into the macrosocial context where the illness originated.”

Again, Foot (2015) provides myriad examples: Alongside patients that were transferred to open community houses or given a “guest status” (which made them free to leave or return to the asylum whenever they wanted), neighbors, families, artists, psychiatry students, and social and political activists flooded into Trieste’s San Giovanni asylum during the last months before its closure. Wards were vacated of patients and converted into workshops in which the ex-patients joined with other people to engage in artistic projects. As exemplified by the Marco Cavallo performance, in which “a large blue papier mâché horse […] constructed in an artwork within an ex-ward, was wheeled through town by patients, artists and activists,” Trieste’s asylum became an experimental, countercultural environment that energized the fibers of democratic life in unparalleled ways. This was not Gorizia in 1968, said Foot (2015: 354), but “Gorizia *as* 1968.” Not unlike the hippie communes to which the institutions of antipsychiatry have often been compared (Crossley, 1998: 885; Schatzman, 1969: 291), or the *halfway houses* used by the Civil Rights Movement (Morris, 1984: 139), Trieste and Gorizia were “working utopias” that, because they were real, “symbolized the realistic nature of [countercultural] movement aspirations” (Crossley, 1999: 826). Activists looked in them for models or frameworks of experience that that the post-revolutionary society would make permanent and universalize (Chapman, 2020b: 4; McGeachan, 2014: 293). Actually, not only the environment of San Giovanni asylum but the entire city of Trieste seemed amenable to transformation at the hands of these teams of inmates, ex-inmates, artists, workers, and activists who peopled the asylum, and many of whom came from other Western countries like the United States. Viewed as adult-run Deweyan school occupations, these initiatives seemed set out to change the future and also the entire scope of material reality. While they originated at the restricted context of a hospital’s undoing, they brought together a variety of peoples, imaginations, and capacities whose freedom, energy, and communication were affirmed over and against the barriers that had originally separated them from each other and from the rest of the world and Italian society (Basaglia & Basaglia Ognaro, 1969: 6). As they crossed these fallen barriers, these democratic projects affirmed the human translations of the dynamics of the life creature; that is, the potential of human association, ingenuity, effort, and imagination to transform the world in ways that brought reality to heightened (if short-lived) levels of meaning and beauty.

### Laing’s Affirmative Dialectics

Skeptic as Laing remained about the possibility of any one institution triggering social change through experiments wrought within it (Basaglia & Basaglia Ognaro, 1973 [1971]: 121), British antipsychiatry conceived the relationship between society and their own mental institutions not in terms of a *negative*—like Basaglia—but rather of an *affirmative* dialectic. Whereas Basaglia negated, Laing made selective affirmations whose democratic impact he pushed to the uttermost consequences. To this extent Laing also followed more closely Dewey’s logical argument; the latter had found its paradigmatic form in schools that selectively imported from society those situations which afforded rich and creative forms of interaction with the environment, yet only to affirm their democratic quality beyond the narrow margins that society had originally set for them, thereby turning them into “allies of art and centers of science and history” (Dewey, 2001 [1915]: 13). While it is true that the Philadelphia Association most often conceptualized the purpose of its institutions in terms of creating safe environments—Laing kept referring to them as “a haven, a sanctuary, a shelter” (Evans, 1976: 158)—I wish to integrate this perspective into the wider Deweyan project. By doing so my analysis will hopefully reveal that providing a safe haven was only one side of the strategy through which the first generation of antipsychiatry’s community households, especially Kingsley Hall, appropriated society’s resources to organize democratic, creative, and therapeutic life experiences for their inmates.

This *experimental*—as opposed to *safety*—dimension of the institutions of British antipsychiatry manifested itself in the range of resources they had to draw on to fulfill their purposes. In consonance with Laing’s and Cooper’s early assimilation of Sartre’s construction of aggressiveness as the interiorization of scarcity (Wall, 2019: 123), *abundance* of space, people, ideas, and objects was an instrumental factor to how these antipsychiatric institutions addressed schizophrenia and psychosis. Preoccupation with spatial and material resources had already oriented Laing’s early experiment at Gartnavel Hospital with the Rumpus Room, and according to McGeachan (2014) it never abandoned him throughout his career. No wonder, then, that three-storied Kingsley Hall, which counted more than a dozen rooms, two halls, three bathrooms, a roof garden, a spacious kitchen, and even a small chapel, qualified as the perfect premises for putting his new ideas into practice (Barnes & Berke, 2002 [1971]: 215–17). In *The school and society*, Dewey (2001 [1915]: 45–54) had envisioned the ideal school as a combination of home, laboratory, factory, studio, library, and a central museum; and we have seen how Addam’s Hull House was all of these things (and many others) at different times. The same can be said of Kinglsley Hall. However, apart from the basic facilities needed to provide food, warmth, and shelter to people who came in under intense mental distress, the institutions of British antipsychiatry also felt the need to draw upon the resources of London’s (counter)cultural scene (Crossley, 1998), upon the “experimental drama groups, avant-garde poets, artists, musicians, dancers and photographers, social scientists of the New Left, classes from the Anti-university of London, and leaders of the commune movement” who regularly congregated at community centers like Kingsley Hall (Schatzman, 1969: 301). What the occupations of industrial capitalism were for Deweyan schools, the sixties’ counterculture was for these community households. This explains why Adrian Laing (1996: 110) conveniently referred to Kingsley Hall as a “refuge”, yes, but one “for leftwingers, radicals, poets, philosophers and people who fell under the all-embracing term ‘artists’.” It was within this milieu that madness found the resources to transform itself. Chapman (2020a) conceptualized Kingsley Hall as both a “single building art colony” and a “countercultural experiment”. “For Laing,” he stated, “madness played out in a supportive experimental community might be a fruitful and therapeutic response to alienation” (2020b: 14–15).

Hopefully the following example will prove this point. In Goffman’s (1991 [1961]: 269) *Asylum*, there is a striking passage in which the author described how inmates who were held in absolute material, immaterial, and social deprivation—with no one to talk to, “naked, and without visible means of expression”—sometimes wrote and drew with their feces on the walls of their cells. Goffman argued that rather than seeing this as the reaction of a desperate individual affirming his or her voice in the face of complete dehumanization (and, therefore, as a positive response that prefigured potentially healthier and richer interactions), asylum staff members took this behavior to be “in keeping with the kind of person who warrants seclusion.” In other words: they distorted the phenomenon to conform to and further legitimize their “disciplinary use of medical practice” (332). By contrast, when Mary Barnes (also in the middle of a psychotic breakdown) spread her excrement on the walls of her room at Kingsley Hall, Laing’s team did not interpret this as madness, let alone symptoms of mental illness, but rather as an index of expressive zeal, creativity, and valuable human qualities that could be strengthened, if adequately worked upon. “Mary smeared shit with the skill of a Zen calligrapher,” Joseph Berke remarked. “I marveled at the elegance and eloquence of her imagery, while others saw only her smells” (Barnes & Berke, 2002 [1971]: 236). Berke soon provided Barnes with “a round toffee tin of grease crayons” and “a lot of scrap paper” for her to use instead of her feces. “Here, just scribble”, he encouraged her. Next, he took Mary down to the local stationary shop, where he bought for her a huge notebook, water colors, and even more crayons (133–4). From then on, Mary was not only allowed to appropriate the building’s facilities to paint on its furniture and walls—“It is not mad to draw on walls,” Laing reassured her. “Artists all through the ages have wanted to cover walls” (144)—but for some time she also hung her immense paintings from the staircase, effectively turning Kingsley Hall into a museum of herself and her work (134). According to Chapman (2020b: 14), “her art became a part not only of her recovery but of Kingsley Hall’s life.” Meanwhile, other members of the team accompanied Mary to the Tate Gallery, bought art books so that she could familiarize herself with past and contemporary movements (Barnes & Berke, 2002 [1971]: 137), and even brought in professional artists from the countercultural scene (141 and 159). Finally, Laing and Berke encouraged her to register at the local School of Arts, where she socialized with art teachers and students and improved her technique until she felt that her growth as an artist no longer depended upon her continued attendance (137–41). Then she quit. Whether she obtained her Degree or not was of no importance to her or to anyone in the community. By the time of her death at the age of seventy-one, Mary Barnes had hosted several individual exhibitions and was a renowned artist, writer, and cultural icon. “My painting,” she pertinently wrote, “had emerged from black lines and breasts on the walls and paintings in shit, to moving figures and scribble on paper: from undercoat paint and wall brushes, to pencils, crayons, charcoal, poster paint, water colour, and oils” (147). The chapters that she authored make it clear that, to her, everything was therapy and treatment (63–4) until, thanks to art, it finally became life.

In one particular issue, however, the institutions of antipsychiatry clearly distanced themselves from Dewey’s philosophy. Being a psychoanalyst himself, Laing fully incorporated the *unconscious* psychic domain into his own work, something that Dewey did not do; for Laing, interaction did not occur only at the horizontal plane of the social and natural environments, but also along the vertical axis through which individuals constantly grappled with their own unconscious “inner space” (MS Laing L 232). This meant that, in order to sustain the patient through a journey of recovery, *regression* and *acting out* (Laing, 1967) played as important a role as did artistic expression and other forms of experimental life embraced by the counterculture. Both formed part of a continuous and single therapeutic process, as implied by Leon Redler’s description of Kingsley Hall: “We wanted to see if and under what conditions acute mental breakdowns could be followed by healing and/or renewal, and whether long-term mental and social impasses could be alleviated in a non-institutional and potentially creative living situation” (MS Laing 22/15A).While regression and acting out were absent from Dewey’s work, to the extent that they implied specific forms of interaction these concepts are translatable into his naturalist ontology, one which they, in turn, help to complement in a similar proportion to which the institutions of antipsychiatry expanded Dewey’s democratic project. In describing an earlier phase of Mary Barnes’ trajectory, for example, Berke mentioned that she “tried to manipulate her environment (by regression) so that she was taken care of in a way which could be identified with an intrauterine experience” (Barnes & Berke, 2002 [1971]: 349). To this aim, the team not only had to feed her milk in a bottle and care for her constantly; they also gave her access to “the Box” (96), a wooden structure “with coloured lights inside” that rested in the basement. Kingsley Hall’s community put itself at Mary’s disposal, not only while she appropriated painting as a technique that allowed her to interact in a more sophisticated and conscious manner with both the outer world and her inner space, but also to get her cleaned and nourished during these regressive lapses. As a result, while Mary Barnes’ trajectory was often used as a confirmation—even an embodiment—of what Chapman (2020b) called the “template of madness as a voyage” that Laing developed after 1967, the enabling role that artistic forms of experience and the entire team at Kingsley Hall played in her development suggests that it can best be understood as a ripple within the wider spiral of growth and of Dewey’s democratic project.

## Conclusion

By analyzing the contributions of Italian and British antipsychiatry through the lens of Dewey’s philosophy, this article has opened paths for reciprocal enrichment. Either as moments of the wider dialectics whereby mental institutions negated themselves as part of an oppressive society, or as real albeit isolated instances of human growth that deserved to be affirmed in themselves, Basaglia’s and Laing’s experimental institutions were radical realizations of Deweyan democracy as much as they remain anticipations of the kind of society and psychiatry that are still worth fighting for. Through its focus on a marginal and extreme phenomenon like schizophrenia, antipsychiatry unearthed the conditions that every single human being needs in order to live a significant and heightened life. Sadly, not only did Laing and Basaglia expose that democratic societies failed to meet these conditions inside their mental hospitals, but that this failure was a result of the lack of these conditions in Western civilization as a whole. To the extent that they were not sufficiently democratic, those societies were ill. Each of them embraced a different strategy to confront this situation, but their efforts bore witness to Dewey’s clear perception that it is only by endlessly pressing for more democratic conditions within mental institutions that psychiatric professionals can boost their therapeutic potential; it is by endlessly pressing for more direct access to material, immaterial, and human resources, and by endlessly encouraging their patients to slowly move through the full circuit of growth (from regressive to artistic and creative forms of experience) that one attains actual wellbeing. While most often read from an educational perspective, Dewey’s (2008d [1939]: 226) vision of “democracy as a way of life” also called for mental institutions that—like those of antipsychiatry—were therapeutic in a *preventive* and a *healing* sense, to the extent that they offered individuals a continuum of rich and worthwhile life experiences. “Might not the lesson be,” Franca Ognaro Basaglia (1985: 18) asked herself, “that disease is a product of the inertia that accompanies every minute of our lives and impedes each and every process that might enable human beings to regain possession of their experience and relationships and to reestablish their dominion over things?”.

From a strictly psychiatric perspective, the institutions of antipsychiatry not only expanded Dewey’s understanding of health, illness, and the circuit of growth; they also completed his grasp of the strategies that led to the kind of artistic and communitarian consummations of experience that Dewey’s entire democratic philosophy aspired to. Mary Barnes’ story suggests that Laing’s team at Kingsley Hall did nothing different from what Dewey expected school teachers to do when he encouraged them to socialize children within the artistic traditions of humanity, so that they could render their own complex experiences into communicable form. With most of the inmates of these community households, the problem was that they had to undergo regressive stages before they could engage with these artistic (or scientific) processes. Laing’s circuit of madness and metanoia—of ego-dissolution and ego-reconstruction—coincided with Dewey’s circuit of growth once the unconscious was included in it as an inner *locus* that forced individuals (as divided entities) to continuously interact with themselves through regression and acting out. To that extent, the institutions of antipsychiatry shared Dewey’s democratic project, and Gorizia’s and Trieste’s asylums, Kingsley Hall, and the rest of community houses proved to be therapeutic since they implied the essential tenets of livelihood itself. In a historical context like ours, where neoliberalism expropriates most of the resources that people need to devise significant life projects and lead them to fulfillment, the institutions of antipsychiatry offer examples of the kind of reconfigurations of material, immaterial, and human resources that make democracy possible amongst illness and precariousness. Like other radical institutions of the sixties and Dewey’s idea of democracy itself, antipsychiatry does not speak to us from the past; it summons us towards an unrealized democratic future.
